# Computational Analysis of Mechanical Properties in Polymeric Sandwich Composite Materials

**DOI:** 10.3390/polym16050673

**Published:** 2024-03-01

**Authors:** Robert Kohar, Jaroslav Miskolci, Lukas Pompas, Lubos Kucera, Peter Stevko, Michal Petru, Rajesh Kumar Mishra

**Affiliations:** 1 Department of Design and Machine Elements, Faculty of Mechanical Engineering, University of Žilina, Univerzitna 8215/1, 02401 Žilina, Slovakia; miskolci@stud.uniza.sk (J.M.); lukas.pompas@fstroj.uniza.sk (L.P.); lubos.kucera@fstroj.uniza.sk (L.K.); 2 Vision Consulting, s.r.o., Československej Armády 732, 93521 Tlmače, Slovakia; stevko4@stud.uniza.sk; 3 Department of Machine Parts and Mechanism, Faculty of Mechanical Engineering, Technical University of Liberec, 46001 Liberec, Czech Republic; michal.petru@tul.cz; 4 Department of Material Science and Manufacturing Technology, Faculty of Engineering, Czech University of Life Sciences Prague, Kamycka 129, 16500 Prague, Czech Republic; mishrar@tf.czu.cz

**Keywords:** sandwich composite materials, polypropylene, polyester, glass, cotton, FEM analysis

## Abstract

This article focuses on the computational analysis of sandwich composite materials based on polypropylene, polyester, glass, and cotton fibers. In the automotive components prepared from these fiber materials, the various components are used in different proportions. Through the manufacturing process, isotropic materials become somewhat anisotropic. Part of this article is aimed at obtaining input values of material characteristics for calculations using finite element analysis (FEM) and the comparison of experimental results with FEM-based material models created using the Digimat 2023.1 software. This article analyzes the modeling of two-phase as well as multiphase composite materials. This work focuses on calculations using FEM according to the test defined in the PR375 standard for loading the finished product in the luggage compartment of a car. The defined methodology enables the application of the FEM-based calculation directly to the product design in the initial phase of research. The construction and production of expensive prototypes and the subsequent production of automotive parts is replaced by computer-based simulation. This procedure makes it possible to simulate several optimization cycles over a relatively shorter time. From the results of computational simulations, it is clear that materials based on PP/PET/glass fibers show a much higher modulus of elasticity than materials created using cotton, i.e., materials of the PP/PET/cotton type. In order to achieve a high strength and stiffness, it is, therefore, appropriate to use glass fibers in the composite materials used for such applications.

## 1. Introduction

This article focuses on sandwich composite materials based on polypropylene, polyester, glass, and cotton fibers. In the production of composites from these materials, their combinations are used in varying proportions. Through the manufacturing process, isotropic materials become partially anisotropic. As a result, methods applicable to the calculation of material properties using common methods show deviations as high as 50% [1,2,3,4,5]. These results are unusable for further construction operations and for defining and designing the components. Due to the current energy crisis and environmental concerns to reduce the emission of CO_2_ in the automotive sector, obtaining a suitable lightweight structure while maintaining the same strength characteristics is very important. This research deals with the composition of sandwich composite materials used in automotive components, e.g., the luggage compartment [6,7,8]. Based on the fibers’ material properties, their interrelationships for the formation of composite laminate and the mutual combination of different types of materials during the creation of the final product are described in this article. This research is aimed at obtaining input values of material characteristics, e.g., tensile, bending, and related properties, for FEM calculations [9,10,11,12]. The FEM-based models were developed and verified with experimental data. In order to have reliable results for sandwich composites, the parameters of FEM calculation must be similar to the parameters of the experimental samples [13,14,15,16]. Research focusing on the characteristics of composites through techniques that analyze the representative volume element (RVE) is known as research using a homogenization technique [17,18,19,20]. The Eshelby model served as the primary tool for the development of widely employed analytical and homogenization methods [21,22,23,24]. For the purpose of modeling the investigated composite material, these methods are insufficient due to significant simplification of the RVE geometry. For a sufficiently accurate description, it is advisable to use the 3D representative volume element (RVE) model of the composites material through finite element method (FEM) tools to obtain the material properties of the composites [25,26,27,28,29]. The RVE can properly describe the characteristics of the whole microstructure using FEM tools such as Digimat or Ansys [30,31,32].

This research work focuses on FEM calculations according to the test methods defined in the PR375 standard [33] for loading the finished composite product in the luggage compartment of a car. The PR375 standard determines the stiffness of the composite product by loading a polymeric composite cylinder with a spherical head with radius 20 mm perpendicular to the composite specimen with a linearly increasing force up to 100 N. Stiffness is determined from the detected deformation at the measured point. The defined methodology enables the application of FEM calculations directly to the design of composite specimens in the initial phase. The obtained results are in the form of tables with comparisons of measured results and FEM-based calculated values. The uniqueness and novelty of the work lies in the development of models predicting the performance of sandwich composites used in automotive parts with a relatively high level of accuracy. The motivation was to develop this model by taking into account the hybridization of synthetic and natural fibers in the reinforcement of composites, which is not so common, and little has been reported in this regard.

## 2. Materials and Methods

There are essentially three steps to producing a finished product from a sandwich composite material. The first step is the production or acquisition of the primary materials, i.e., the fiber materials necessary for the production of composite laminates. This mainly concerns the composition of the material, its preparation for production, as well as the actual production of the fibrous structure. The second step is the production of the composite laminate itself by combining the raw materials from the first step. The last step is the preparation of the sandwich composite material, combining various composite laminates from the second step and inserting foils and paper-based honeycomb structures. The advantage of sandwich composite materials is the theoretical possibility of using up to 100% recycled materials in the production of composite laminates. The quality of the basic fiber materials and the subsequent processing of individual laminates in the preparatory and final processing steps have a significant impact on the strength of the final product itself.

### 2.1. First Step: Preparation of the Input Material

The input material can be divided into two groups. The first group is organic materials (cotton, kenaf fibers). The second group is inorganic materials: polypropylene, polyester, glass fibers, and their recycled semi-finished products. Their mutual combination creates composite laminates in the input production process. The natural fiber material, kenaf, is obtained from the stems of the hemp hibiscus called *Hibiscus cannabinus* in Latin. Its use in the textile industry was first recorded in the 18th century. It entered the automotive industry at the turn of the millennium as a replacement for glass fibers. It is not possible to use kenaf for exterior components. It can only be used for interior parts as it has the ability to absorb moisture, which can result in the formation of mold.

The cotton used in the production of composite laminates is obtained from old clothing or from waste generated during the production of clothing. The input material is torn into the fibers in a roller shredder.

**Figure 1:**
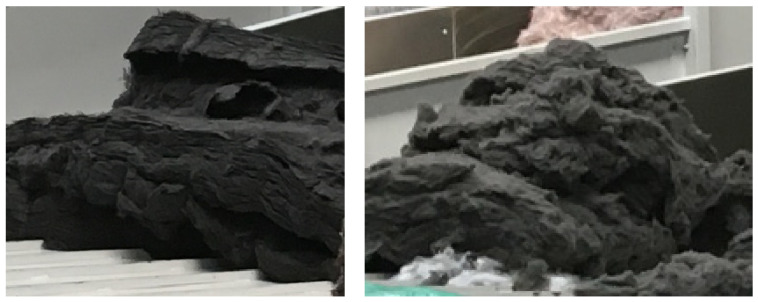
Polypropylene PP (**left**) and polyester PS (**right**).

Glass as a material has been known since around 4000 BC. It was created as a byproduct of the metal industry. Currently, it has become the basic raw material for the production of glass fibers, so-called E-glass, which contains 55% SiO_2_, 18% CaO, 8% Al_2_O_3_, 4.6% MgO, and other elements with a share of less than 5%. Glass is used in exterior components due to its resistance to moisture; its disadvantage compared to kenaf is the fact that its manufactured components are heavier. Polypropylene and polyester in the form of loose fibers are used in the production of composite laminates and components. Figure 1 shows polypropylene and polyester ready to be fed into a mixer.

### 2.2. Second Step: Preparation of Composite Laminates

The preparation of composite laminates begins in the feeder machine, where individual raw materials are dosed at the input. Their quantity is weighted to achieve the required ratio of fiber fractions. Next, these raw materials are moved to a mixer. This process is very important to achieve the homogeneity of the material. In the later process of heating and shaping, the process parameters are defined based on the mutual ratio of the input raw materials.

**Figure 2:**
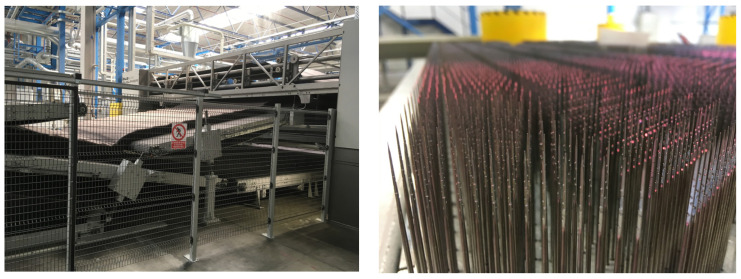
Semi-finished product layered in several layers (**left**) and detailed view of the needles (**right**).

The next step in this process is to create a simple nonwoven carpet. According to the requirements for the nonwoven process, the semi-finished product is layered in several layers (Figure 2 left). This process increases the base weight of the composite laminate. In this process, if necessary, resins can be added, which come from recycled materials and were created as waste during the trimming of the molding. In the last step, this nonwoven material is punctured several times (Figure 2, right). Through this process, selected fibers are oriented in the thickness direction, and layers are connected and condensed. Such a bonding technique is known as needle punching, and this enhances the mechanical stability of the product. There is no damage to fibers due to the special geometry of the barbed needles used in nonwovens process. The material prepared in such a way still needs to be cut to the appropriate dimensions and is ready for the shaping process.

### 2.3. Third Step: Preparation of the Finished Product from Composite Laminates

The input material is composite laminate. The first step is to prepare the sandwich material on the worktable. Here, the produced composite laminates are bonded with other layers in the sandwich. These are mainly insulating films, foils, paper honeycombs, etc., used for the production of luggage compartment and floor components in an automotive.

**Figure 3:**
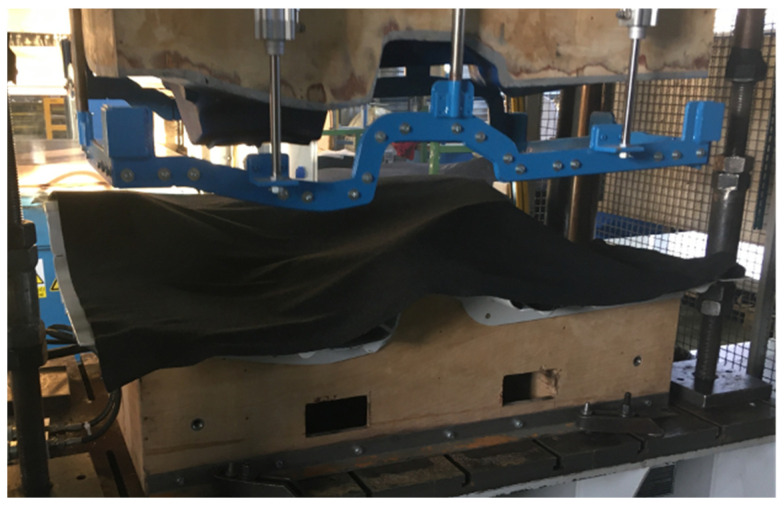
View of the production of composite material for the luggage compartment of a car.

The next step is to insert the sandwich material into the heating press. Here, it is heated and compressed to the required thickness. This process is a very important step to achieve the desired mechanical performance. If the heating time is too long, overheating may cause burning of the PP component and, thus, insufficient bonding/impregnation of the individual fibers. In the same way, a short heating time will not achieve suitable melting of this PP component, which will cause the separation of the individual layers. The next step is transferring the already prepared sandwich material from the heating press to the forming press. This operation is performed manually or automatically. Here, it is important to stay within the maximum time for this action so that this material does not cool down excessively. Said time is in the order of a few seconds only. The semi-finished product in the molding press is shown in Figure 3.

After this semi-finished product has been pressed and cooled, it is subsequently pulled out of the pressing tool. The pressure used is in the range of 20 to 200 tons depending on the size of the pressed semi-finished product. The tool is cooled to about 20 °C; the inserted blank has a temperature of about 220 °C and is formed and cooled for about 90 s. This semi-finished product is then cut with a water jet or with a cutting tool. The waste after trimming is cut up and used again in the production of composite laminates.

### 2.4. Modeling of Composite Materials

This concerns heterogeneous materials whose microstructure consists of a matrix material and multiple phases called inclusions, which can be short fibers, platelets, microvoids, or microcracks. There are several examples of such composite materials, such as the following: thermoplastic polymers reinforced with short glass fibers (GFRP), rubber matrix composites (RMC), metal matrix composites (MMC), such as titanium reinforced with carbon or ceramic fibers, concrete reinforced with short steel fibers, and biomaterials. The goal of micromechanical modeling is to predict the interaction between the microstructure and the macroscopic (or overall) properties [34,35,36].

Consideration is given to a heterogeneous body whose microstructure consists of a matrix material and multiple phases called inclusions, subjected to specific loads and boundary conditions. The goal is to predict the influence of the microstructure on the body’s response. It would be computationally infeasible to solve the mechanical problem within the scope of the microstructure. Therefore, two scales are distinguished: microscopic (extent of heterogeneities) and macroscopic, where the body can be considered locally homogeneous.

The link between these two approaches is established through the concept of the representative volume element (RVE). At the macro scale, it is assumed that each material serves as the center point of an RVE, which must be sufficiently large to represent the underlying heterogeneous microstructure and, at the same time, small enough when compared to the overall size of the component. A two-step approach facilitating the transition between the micro–macro approach in both directions can be summarized in the following steps:

1. macro material point: center of the representative volume element (RVE);
2. on a microscale: the RVE contains a finite number of components;
3. a constitutive model is needed for each of the components;
4. micro/macro transition: homogenization method to find the macro-constitutive response of the RVE;
5. macroscale continuum mechanics with a macro-constitutive equation. Macro–micro transition: At each instant and at each point of the macro-material, a numerical approximation is made to see what is happening at the microscale (e.g., stresses and strains in each phase) [34,35,36].

The basic problem of homogenization in the field of linear elasticity is to find an equivalent homogeneous material that has the same effective macro-stiffness as a real heterogeneous composite under the same boundary conditions. There are several methods of homogenization that solve this problem: asymptotic or mathematical theory of homogenization, cell and sub-cell analysis methods, field transformation analysis, direct finite element analysis, and field homogenization. The latter two approaches can be used in the Digimat software package.

Several software tools can be used to estimate the properties of composite materials, such as Ansys, Abaqus, MSC Digimat, etc. This work deals with the description of a software package that is licensed at the University of Žilina and with which it is possible to model composite materials.

Digimat solutions are used by CAE engineers, material scientists, and composite material manufacturing process specialists to accurately predict the nonlinear micromechanical behavior of complex multiphase composite materials and structures (PMC, RMC, MMC, nanocomposites, etc.).

**Figure 4:**
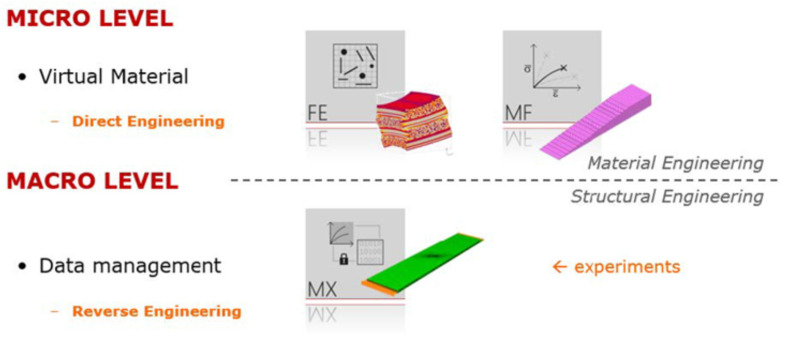
Possibilities of using Digimat tools [35].

The Digimat platform offers its users three different categories of products:

- Tools: A set of interoperable software products aimed at professional use for material or structural engineering purposes (MF, FE, MAP, CAE) (Figure 4).
- Solutions: Use of Digimat technology in a fully integrated GUI-controlled environment for specific tasks (RP, VA, AM).
- Expertise: Knowledge transfer from 10+ years of experience in micromechanical modeling. It includes a documentation file and a collection of examples.

The Digimat platform provides access to a suite of software tools that allow for the modeling of the nonlinear anisotropic behavior of composite materials and for predicting structural properties using advanced material modeling. Material models obtained using MF and FE tools can be stored in the MF material library along with additional information, such as real material tests, supplier information, etc. For FEM calculations, it is possible to export material models for FEM software such as Ansys Workbench 2023 R1, Marc Mentat 2023.1, DS SIMULIA Suite 2023, etc., using this module. Another option is to obtain material characteristics from commercial companies through the MX module [36].

### 2.5. Design of a Composite Part for a Luggage Compartment

**Figure 5:**
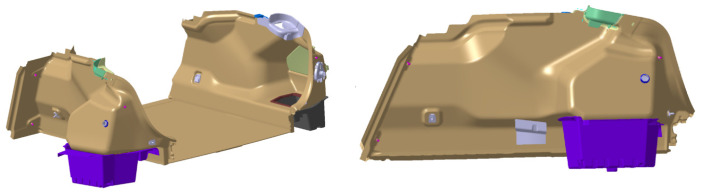
Design of the luggage compartment made of composite materials.

A modern car also includes a functional luggage compartment. The following section describes the design of the part of the luggage compartment that is made of composite material, the production of which is described in Section 2.1, Section 2.2 and Section 2.3. The individual parameters of functionality and testing of the luggage compartment lining are defined in the design specification for the given product. After the finalization of the initial design, further analyses of the component are carried out. The conditions for manufacturability, static and dynamic loads, and the analysis of the design under climatic test conditions are analyzed. Figure 5, on the left, shows an overall spatial view of the design of the luggage compartment created in this work. A detailed view of the component is shown in Figure 5, on the right.

**Figure 6:**
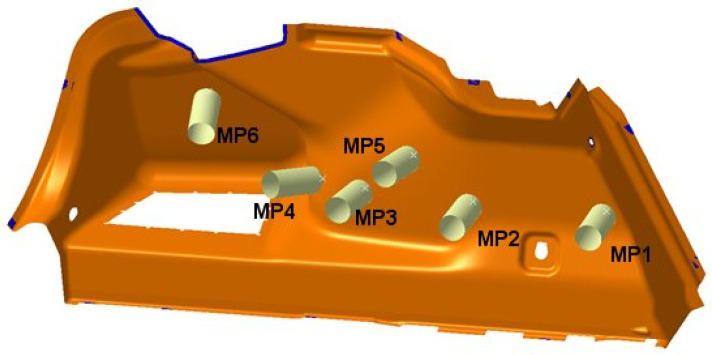
Determination of measurement points.

For the side panels, one of the main points of analysis is the resistance of the component to the prescribed load of 100 N according to test defined in the PR375 standard. Since the design of the component must contain all visible surfaces of the luggage compartment, the structural design in the initial phase must divide the surfaces into individual components and separate the surfaces for the composite parts from the surfaces for the side lining of the luggage compartment, the storage space, the anchoring elements, and the fastening parts. The next stage is the determination of the correct ratio of material composition, the amount of material, the manufacturability of the component, and, of course, the cost calculation for the recommended material. To determine the right material, verification of the design and its geometry is important. Considering that the final test always takes place perpendicular to the specimen at the location of the test point, this requirement for the selection of the test point must take into account the spatial requirement for the physical testing of the component. The defined measurement points are shown in Figure 6. Considering the technical parameters, optimal FEM calculations are carried out.

In order to define lightweight composite materials, it is necessary to achieve the lowest possible bulk density. The density of polymers is generally around 1 kg/dm^3^. Suitable materials for applications in a luggage compartment are polypropylene (PP) with a density of 0.92 kg/dm^3^ and polyester (PET) with a density of 1.38 kg/dm^3^.

The mutual combination of PP and PET fibers with a fineness around 6.7 dtex results in a material with an areal density between 1000 and 2100 g/m^2^ and a density after molding of around 0.5 kg/dm^3^, depending on the resulting thickness of the material, where the PP component becomes the matrix, and the PET becomes the reinforcing component. This is due to the fact that the melting point of PET is 260 °C, and PP melts at 220 °C. The mutual combination of these two components creates an optimal luggage compartment.

### 2.6. Tests for the Experimental Verification of the Calculated Values

The first type of tensile test was conducted on an Instron 8862 testing machine equipped with a non-contact deformation measurement system using digital image correlation (DIC) with specimens of a 50 mm width, employing a Canon D40 camera (Canon Inc., Ota city, Tokyo, Japan) and the DIC analysis software ImageJ 1.52i. The output of the deformation measurement was a 2D map of the specimen’s surface. From this map, the deformation values corresponding to the contact sensor (gauge length 100 mm) were extracted, and a stress–strain curve was constructed. In the range of 1000 to 3000 microstrains, the modulus of elasticity was determined. The test specimens were prepared in accordance with the ASTM D 3039 standard [37].

The second type of tensile test involved cyclic loading in the range of 0.2 to 2 MPa for 100 cycles to monitor the elastic response of the material and the evolution of plastic deformation. Regression analysis was used to calculate the range of data from 0.5 to 1.5 MPa. The loading was performed under the same conditions as the first type of test. The test specimens were prepared in accordance with the ASTM D3479 standard [38].

**Figure 7:**
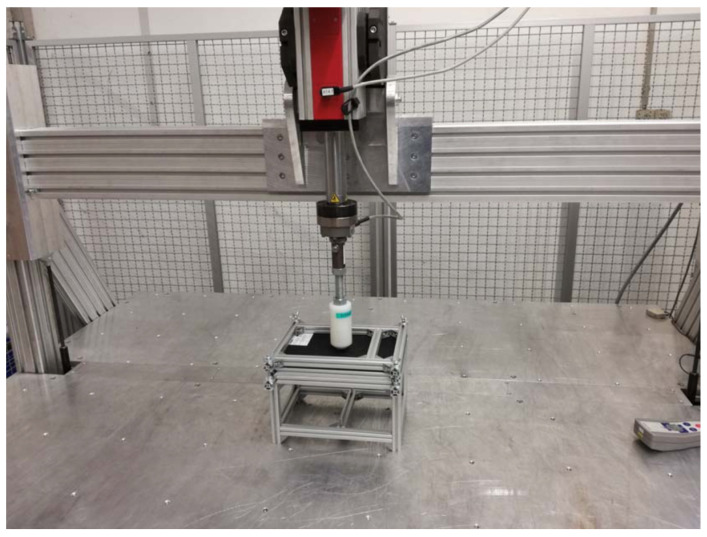
Measuring device for verifying the material’s characteristics.

**Figure 8:**
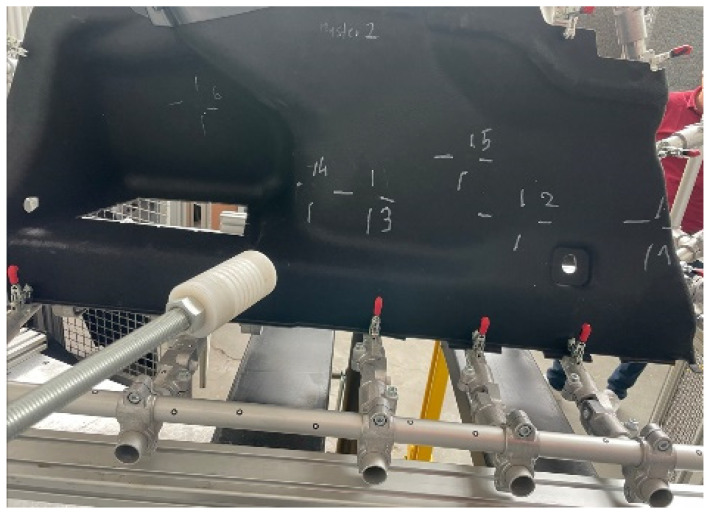
Example of the location of the measuring attachment for various testing points from MP1 to MP6.

For the tests according to the PR375 and similar standards, a measuring device with the possibility for carrying out vertical and horizontal measurements was built (Figure 7). The measuring device was equipped with a controlled force extensometer, Zwick/Roell Z5.0 TS, which was integrated with the measurement software testXpert III. For verifying the calculation, a measuring device was built for clamping the side paneling of the luggage compartment on the left side. A measuring attachment according to the PR375 standard was clamped on the power cylinder. The clamping points of the measuring device were identical to the clamping points in the FEM calculation. To exclude unwanted movements during the measurement, the measuring device was fixed on the measuring table. In order to achieve the required measurement direction, the test fixture with the clamped part and the power cylinder had to be set in a mutually opposite position so that the applied force was always perpendicular to the surface at the testing point. The accuracy of the setup was verified using the portable coordinate-measuring machine FaroArm Platinum. This change in position had to be set for each point separately (Figure 8).

## 3. Results and Discussion

### 3.1. Analysis of the Geometric Surface for a Quality Compartment

For the extraction of relevant geometric surfaces, knowledge of the use of the planned material in the serial production of the surrounding components is required. These are mainly sound insulators made of materials with a very low-volume density that do not provide any mechanical support. On the contrary, firm, unyielding points of fixation are very important. A special group consists of electrical components including cabling, a point in the production process during which it is important to know the thermal radiation emitted by the device and the possible requirement for a minimum distance from this device.

**Figure 9:**
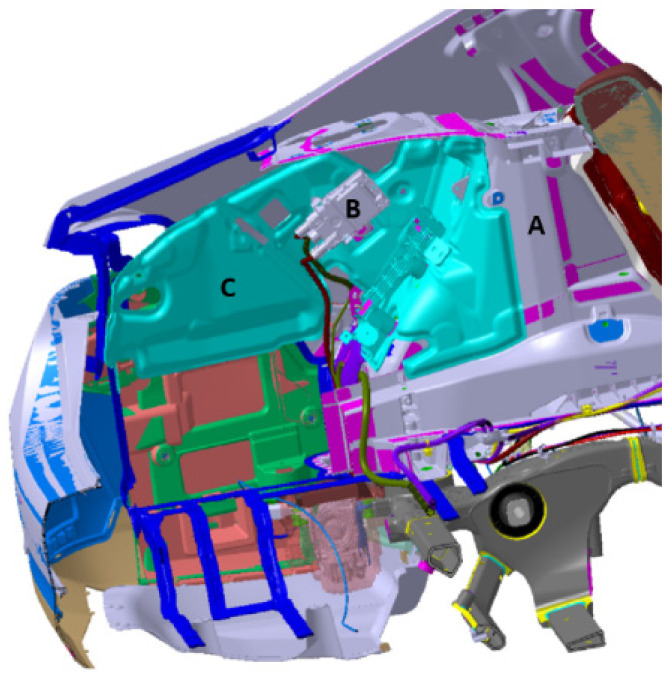
Structural design of the rear part of the car: A—body, B—electronics and cables, and C—polymeric components.

A special group of supporting geometry consists of parts made of polymers or other suitable materials to ensure the required strength of the component. These are also design elements or storage spaces to which access is possible through the side paneling. These polymeric components are easily structurally modifiable. According to the results of the FEM, it is possible to adjust these parts and, in certain areas, provide structural support. This design modification has a favorable effect on the type of material used for the side lining, the weight, and the performance. This change in the design of the polymeric component or other supporting parts is implemented in the geometry of the component. Figure 9 shows a completed component for attaching and fixing the manufactured part in the body of an automotive. After the FEM calculation and the real experimental tests on the manufactured composite component, further design changes can be implemented so that the conditions of the test for the final quality specifications are met.

**Figure 10:**
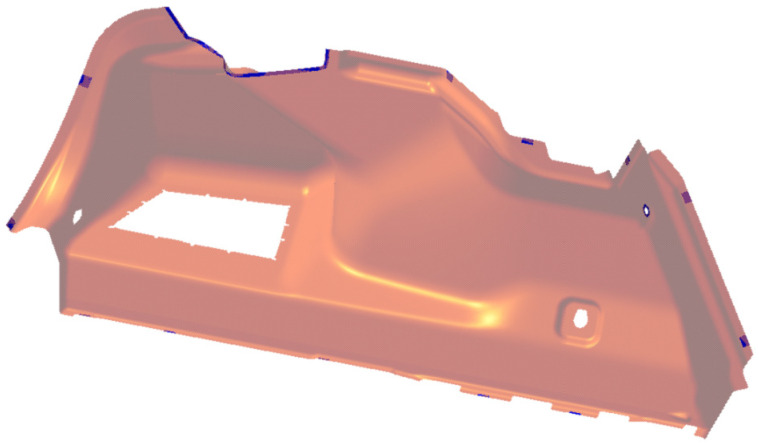
Model in CAD for the luggage compartment of a car.

Before performing the FEM calculation itself, the tested area must be modified for the needs of the FEM software in the following ways:

- checking the connection of surfaces—the design of surfaces takes place in special programs for creating designs such as, for example, Maya/Autedesk, where the output surface must be further modified for the needs of FEM. The most important parameter is the “connexity check” to 0.001 mm. This treatment of the surfaces will prevent the overlapping of the surfaces as well as remove the micro-gaps between the surfaces;
- creating a central zero surface; then, the ability of the surface to offset the required thickness is checked in both directions. Here, it is necessary to replace problematic surfaces with new ones, similar in shape to the original design;
- due to the production technology for the side lining, there is a difference in material thickness. The expected course of the material thickness in the final product needs to be defined for the FEM model. Figure 10 shows the model.

### 3.2. FEM Analysis of the Model for the Luggage Compartment

The preparation and calculation with the FEM model were performed using the Hexagon MSC package in several steps, as described below.

**Figure 11:**
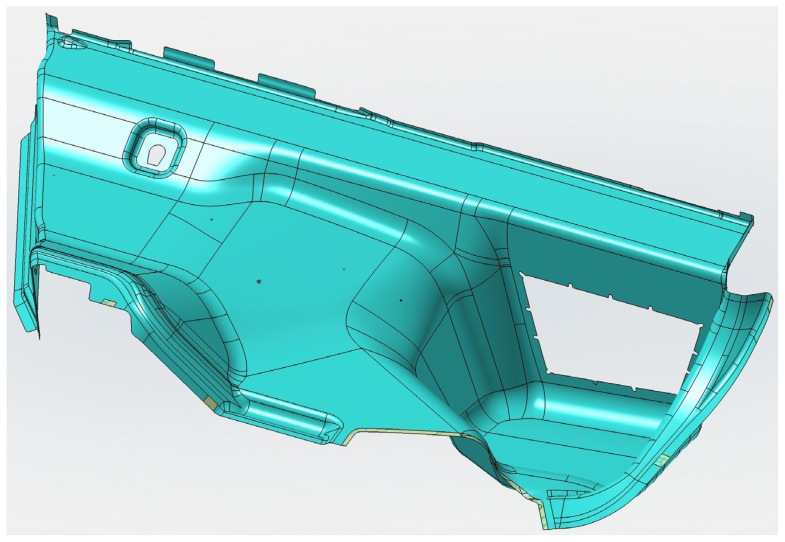
Surface model of the luggage compartment loaded in MSC.Apex.

**Figure 12:**
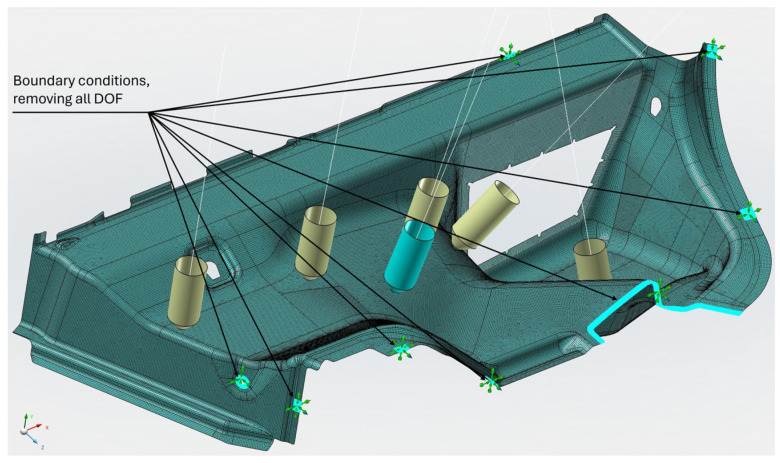
Defining the FEM of the network, boundary conditions of anchoring—all degrees of freedom (DOF) were removed (x, y, z, rot x, rot y, and rot z) and loading in MSC.Apex took place.

**Figure 13:**
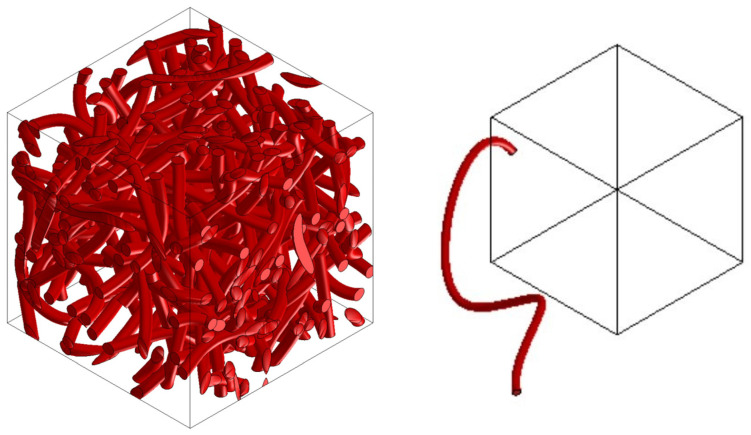
RVE material model (**left**) and shape of an element (**right**).

**Figure 14:**
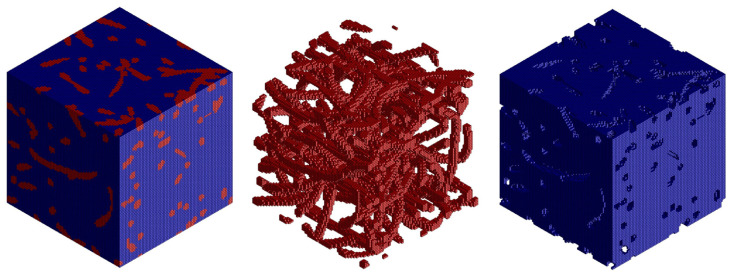
FEM model of the RVE (**left**), FEM model of the inclusions/elements (**middle**), and FEM model of the matrix (**right**).

**Figure 15:**
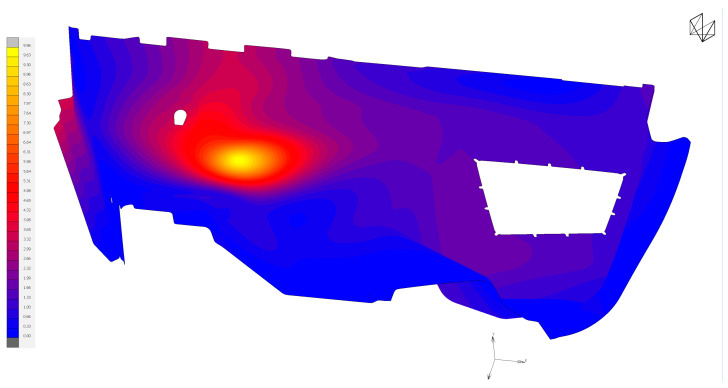
Detected deflection for the MP2 measuring point [mm].

1. Preparation of the FEM model—defining the finite element network and defining the boundary conditions using the MSC.Apex software (Figure 11 and Figure 12).
2. Material preparation was performed using MSC.Digimat FE. The standard material used in the design of these components was a combination of PP/PET fibers in a ratio of 50:50. A material with a thickness of 5 mm and a density of up to 450 kg/m^3^ was designed for this component. After defining the basic material properties (Table 1), the type, shape, and dimensions of the volume elements were defined. Subsequently, the RVE was generated (Figure 13). To obtain the properties of the composite, a finite element mesh of the RVE model was generated using 512,000 elements and 531,444 nodes (Figure 14). Using the function “Automatic properties evaluation”, six loading steps were applied by Digimat FE to the prepared FEM model to obtain all the material properties (Table 2). As a solver, the MSC Marc CASI Iterative solver was selected, and the contact algorithm node-to-segment was applied.
3. The prepared FEM model was subsequently loaded in MSC.Marc/Mentat, where contact pairs, loading states, and material models were defined according to Figure 12. Figure 15 shows the deflection at measuring point MP2.

### 3.3. Experimental Verification of Calculated Values

For the experimental verification according to Section 2.1, Section 2.2, Section 2.3, Section 2.5, and Section 2.6, composite samples were produced for testing. The properties of the basic fiber materials used in the production of the samples are listed in Table 1. The basic materials were not further modified to ensure conformity with the FEM calculation. After weighing and mixing the basic fiber materials, they were prepared into layers to achieve the required density. Subsequently, the semi-finished product was heated to a temperature of 220 °C. After heating, the semi-finished product was transferred to a pressing tool, where it was formed and cooled under a pressure of 180 tons for 90 s. Following this, the formed semi-finished product was cut into its final shape using a water jet cutting tool. After the experiments, samples were taken from the manufactured products and inspected to verify the appropriate heating of the material.

**Figure 16:**
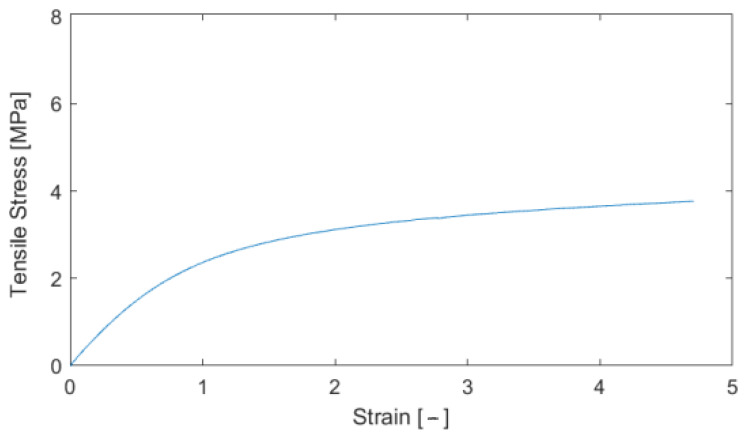
Stress–strain curve for the determination of the modulus of elasticity.

Composite samples were produced for tensile tests. Table 3 shows the results from the tensile tests, and Figure 16 shows the stress–strain curve.

**Figure 17:**
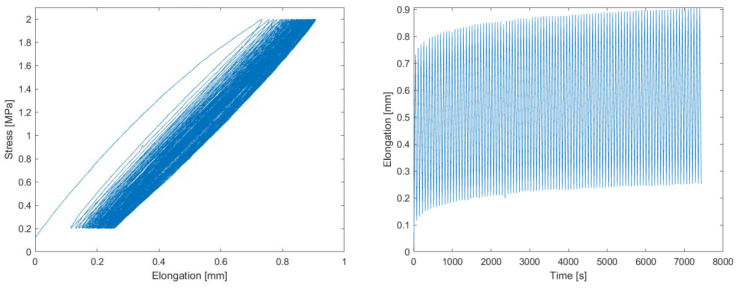
Elongation–stress curve (**left**), and time dependence of the elongation curve (**right**).

**Figure 18:**
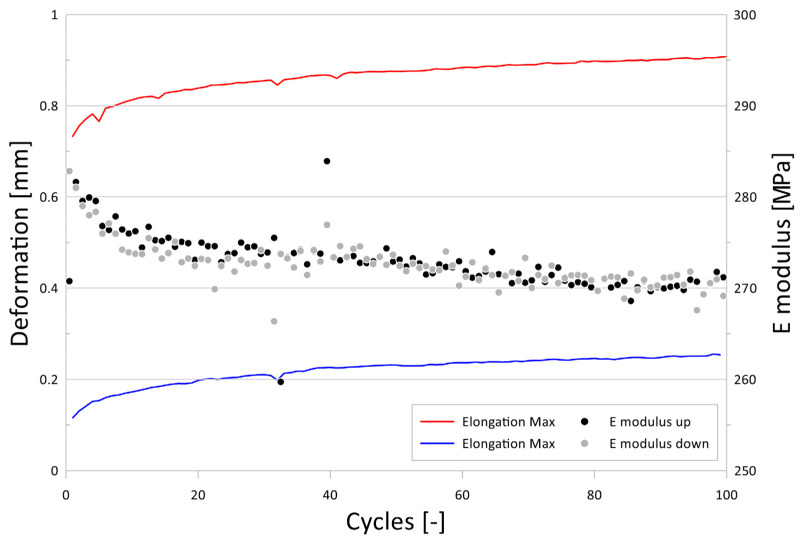
Evolution of the modulus of elasticity determined from the ascending and descending branches during tensile cyclic loading, along with the maximum and minimum elongation values of the test specimen, depending on the applied cycles.

The tensile test with cyclic loading in the range of 0.2–2 MPa was evaluated for each of the 100 loading cycles for both the loading and unloading cycles. In total, 200 values of the modulus of elasticity were obtained for each test specimen. Figure 17 shown an example of stress–strain recording during the elongation of the test specimen and the time dependence of the elongation. Figure 18 shows the evolution of the modulus of elasticity. Within the investigated range of loading (up to 2 MPa), a stabilization of the plastic deformation changes was evident after approximately 20–30 cycles, with the most significant increments in deformation occurring within the first 10–20 cycles. This corresponds to the typical logarithmic behavior of viscoelastic deformation over time. The influence of this deformation on the determined modulus of elasticity was not significant and fell within the dispersion range caused by material heterogeneity. Table 4 shows the results from the tensile tests.

**Figure 19:**
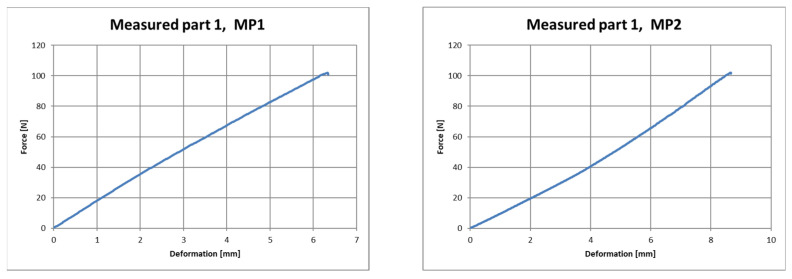
Force–deformation curve during loading for the first measured part and for measuring point 1 (**left**) and measuring point 2 (**right**).

**Figure 20:**
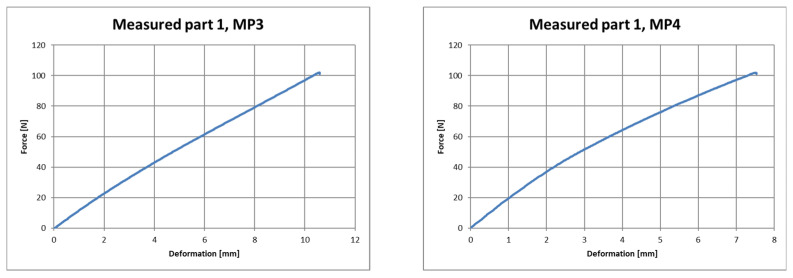
Force–deformation curve during loading for the first measured part and for measuring point 3 (**left**) and measuring point 4 (**right**).

**Figure 21:**
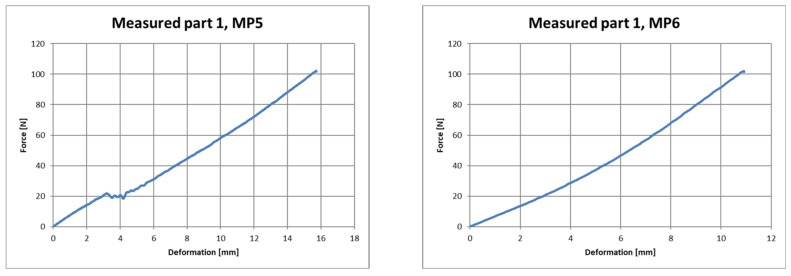
Force–deformation curve during loading for the first measured part and for measuring point 5 (**left**) and measuring point 6 (**right**).

The output of the measurement program utilized was a table with the measured quantities and a graphic representation of the test. Figure 19, Figure 20 and Figure 21 show the course of deformation during loading for the composite component and measuring points 1 to 6. A total of six points on three samples were tested. In order to eliminate changes in the production parameters, it was procedurally ensured that these samples came from the same production batch of polypropylene used as the raw material.

### 3.4. Comparison of Measured Data and Calculated Values

Based on the measurements taken at points MP1 to MP6, the data were processed. For the individual points, evaluations were made in the MARC program according to the material model from Digimat FE. In Table 5, a comparison is made between the measured and calculated deformations for the loading forces according to the PR375 standard.

Based on the analysis of the results (Table 5), it follows that, for measuring points MP2, 3, and 4, sufficiently accurate values of deflections from the FEM calculations were achieved; the difference in the results was up to 15%. Higher differences were found for the remaining points. These were caused by the “frog” effect, where excessive deflection of the material occurred during loading under the action of a relatively small force (Figure 21 left), and the FEM calculation could not predict this effect.

Based on the results described in the above section, using the proposed methodology, it is possible to design composite luggage components with a sufficiently accurate prediction of the properties for the materials used. At the same time, during the experimental verification step, it became clear that, sometimes, the FEM calculation did not take into account certain effects, but the frequency of production of experimental samples can be reduced to a minimum, thereby reducing the total cost.

### 3.5. PP/PET/Cotton Material Design

**Figure 22:**
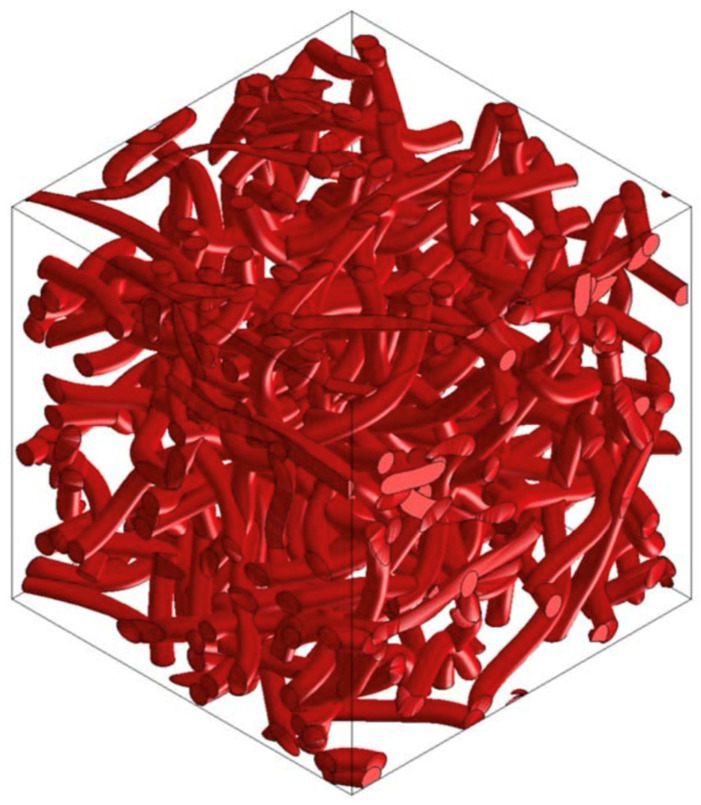
RVE geometry for a composite made from PP/PET/cotton—60/0/40.

**Figure 23:**
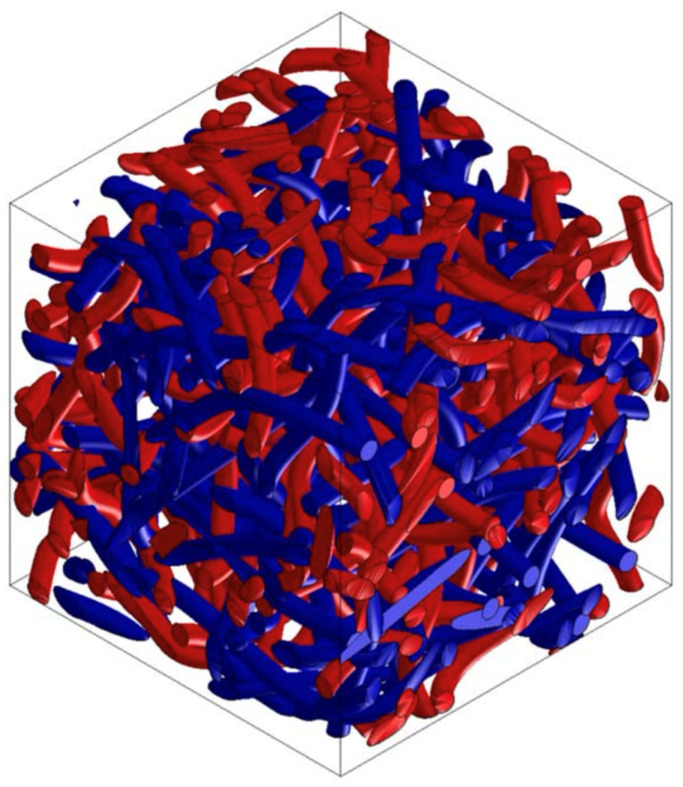
RVE geometry for a composite made from PP/PET/cotton—50/25/25.

From the point of view of renewable resources, even in the automotive industry, manufacturers focus on the use of ecologically recyclable materials. Waste cotton fiber is such a material, which can be used as an additional component or can completely replace the supporting component. Selected versions of the material were analyzed using Digimat FE for the accurate determination of material properties in all directions. Figure 22 and Figure 23 show the RVE geometry with the visualization of the inclusions. Table 6 shows the determined results of the tests on the material properties.

### 3.6. PP/PET/Glass Fiber-Based Material Design

**Figure 24:**
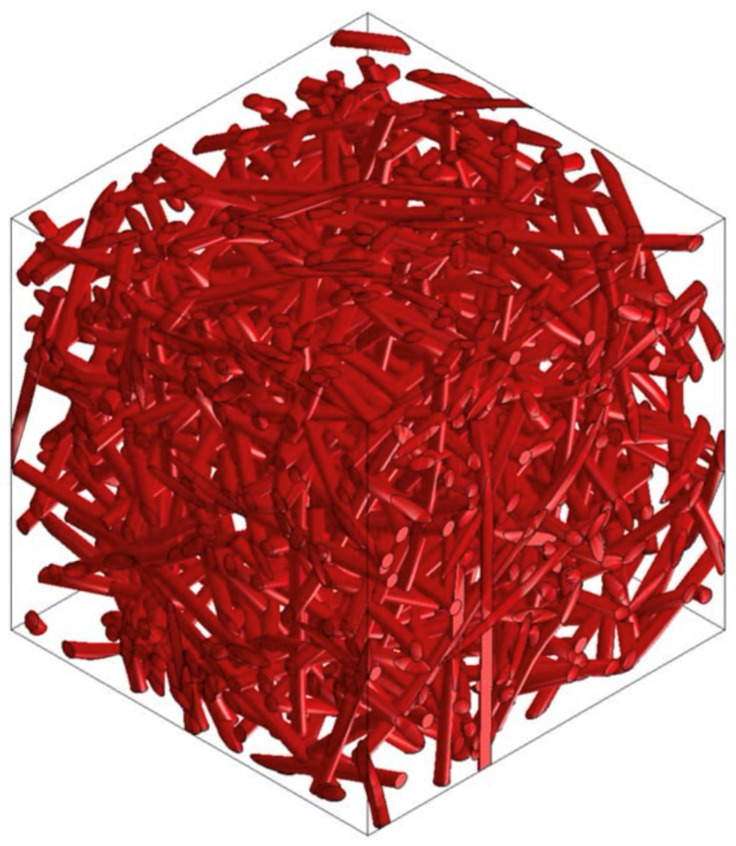
RVE geometry for a composite made from PP/PET/glass fibers—60/0/40.

**Figure 25:**
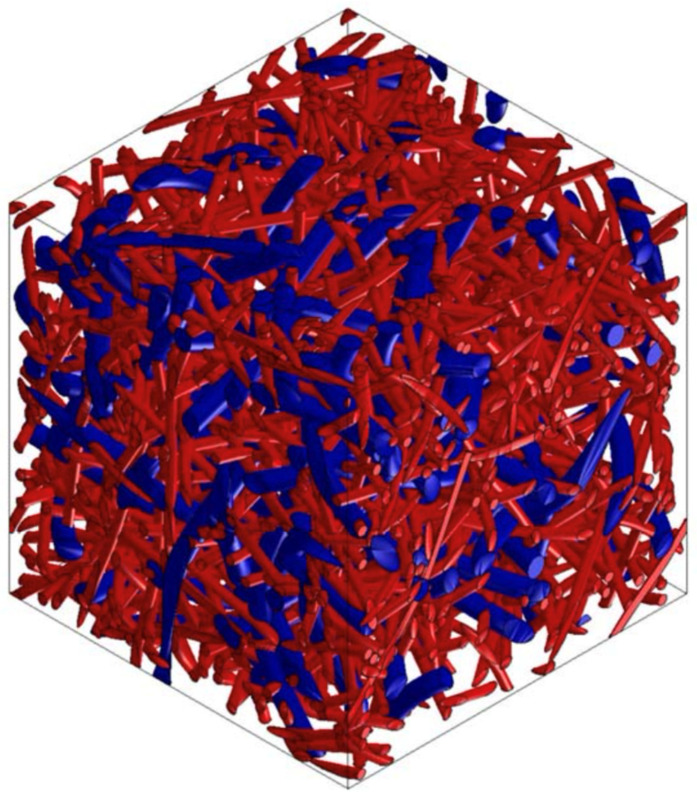
RVE geometry for a composite made from PP/PET/glass fibers—50/25/25.

**Figure 26:**
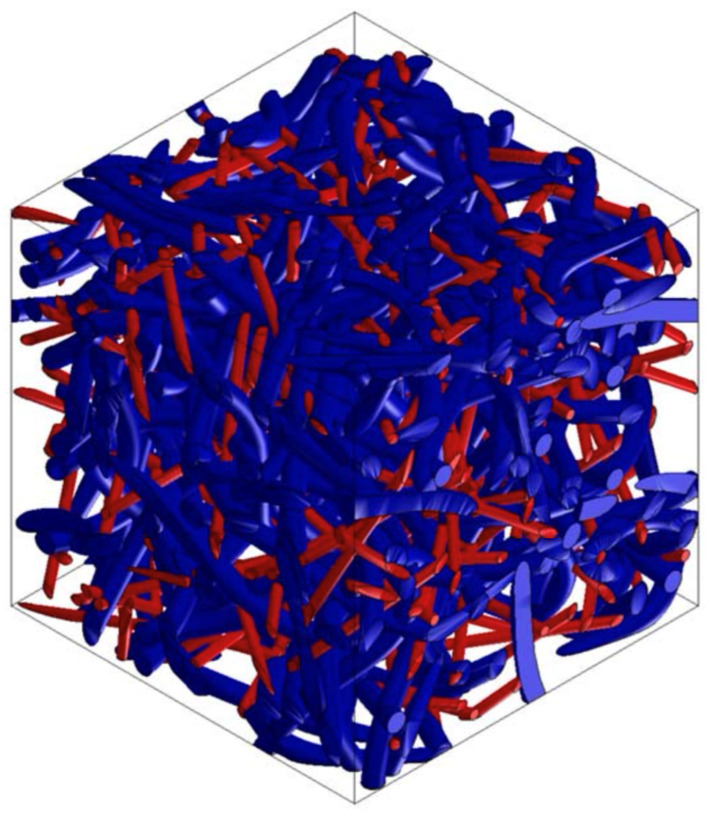
RVE geometry for a composite made from PP/PET/glass fibers—50/45/5.

Other research deals with the design of the composite materials used in the lining of cars, which demands a higher stiffness of the structure. To increase stiffness, it is necessary to use a material that has a higher modulus of elasticity than thermoplastic polyester (PET) and can be used as a supporting element. Glass fibers are suitable for such applications. Selected versions of this material were analyzed using Digimat FE for the accurate determination of their material properties in all directions. Figure 24, Figure 25 and Figure 26 show the RVE geometry with the visualization of the inclusions/fibers. Table 7 shows the results of the tests on the material properties. The analysis of the results shows that glass fibers can be used as a supporting element for structures where an increased rigidity is required.

## 4. Conclusions

This work analyzed the modeling of two-phase as well as multiphase composites. It focused on calculations according to the test defined in the PR375 standard for loading the finished product, i.e., a luggage compartment, in a car. The defined methodology enabled the application of the FEM calculations directly to the design of the components in the initial phase of this work. The construction and production of expensive prototypes and the subsequent production of components was replaced by computer simulation. This procedure makes it possible to simulate several optimization cycles in a relatively shorter time.

The results of the simulation were used as input data for the creation of the design, the construction of the composite components, and the optimization of the surrounding components. The mentioned computer simulations in the Digimat software were used in the design of composite parts for the automotive industry, and it can be possible to use them in other industries where the use of composite materials is expected.

It is clear from this work that Poisson’s ratio was largely very similar for the different materials used. The values of the modulus of elasticity differed significantly according to the type of materials used in different proportions. Accurate values for isotropic materials were obtained from the analysis created with SW Digimat MF. SW Digimat FE is more suitable for creating models of non-isotropic materials. This allows for a more accurate modeling of the actual volumes used within the finite element. With the results of the material model obtained from SW Digimat FE, more accurate results can be obtained, especially when solving spatial loads.

During the above-mentioned phase of this work, the real experimental properties of the materials after reshaping were determined. In the case of complex shapes of individual components, the thickness of a material changes in certain places where the material bends, changing its local mechanical properties. So far, this has been solved by changing the thickness of specific parts of the product. The mechanical properties in our experiment were assumed to remain largely unchanged. Therefore, there were large deviations between the deformation observed during the experimental test and the deformation taking place during the FEM calculation. There will always be a certain deviation when comparing the calculation and the measurement, but the preliminary calculation in our study pointed out the weak points of the construction with sufficient accuracy. It would be ideal to maintain a constant thickness of the product, a characteristic which would also have to be implemented in the production technology. This leads to more complex conditions for the production of molds for defining the final shape of a product.

From the results of our computational simulations, it is clear that materials based on PP/PET/glass fibers have a much higher modulus of elasticity than materials made using cotton, i.e., materials of the PP/PET/cotton type. In order to achieve a high strength and stiffness, it is, therefore, appropriate to use glass fibers. In addition to glass fibers, Kevlar or carbon can also be used, in which case it is assumed that the strength will increase in various directions when used in automotive components.

## Figures and Tables

**Table 1:** Material properties of basic materials.

Material	Modulus of Elasticity E [MPa]	Poisson’s Ratio µ	Density ρ [kg·m^−3^]	Fiber Diameter [mm]	Fiber Length [mm]
Polypropylene PP	1450	0.4	910	0.025	10
Thermoplastic polyester PET	1300	0.4	1300	0.020	10

**Table 2:** Calculated material characteristics.

Material	PP/PET 50:50
Modulus of elasticity E_11_ [MPa]	370.3
Modulus of elasticity E_22_ [MPa]	366
Modulus of elasticity E_33_ [MPa]	386.9
Poisson’s ratio µ_12_	0.287
Poisson’s ratio µ_21_	0.284
Poisson’s ratio µ_13_	0.276
Poisson’s ratio µ_31_	0.289
Poisson’s ratio µ_23_	0.274
Poisson’s ratio µ_32_	0.289
Modulus of elasticity in shear G_12_ [MPa]	139.5
Modulus of elasticity in shear G_23_ [MPa]	141.2
Modulus of elasticity in shear G_13_ [MPa]	142.4
Bulk density ρ [kg·m^−3^]	442.4

**Table 3:** Results from the tensile tests type I.

	a [mm]	b [mm]	L_0_ [mm]	E [MPa]	E_avg [MPa]	E_sd [MPa]
Tensile test type I	50.21	1.78	100	380	373	11.9
	50.02	1.79	100	358		
	50.14	1.80	100	365		
	49.78	1.81	100	371		
	50.09	1.84	100	392		
	49.74	1.82	100	389		
	49.75	1.73	100	361		

**Table 4:** Results from the tensile tests type II.

	a [mm]	b [mm]	L_0_ [mm]	E [MPa]	E_avg [MPa]	E_sd [MPa]
Tensile test type II	50.03	1.84	100	375	375	11.4
	49.74	1.82	100	389		
	49.75	1.73	100	361		

**Table 5:** Comparison of calculated and measured deformations.

	MP1	MP2	MP3	MP4	MP5	MP6
Measured deformation (mm)	5.31	9.53	10.28	6.31	14.49	10.71
Calculated deformation (mm)	3.52	9.96	10.63	4.76	9.49	9.61
Difference [%]	33.71	4.51	3.40	24.56	34.51	9.34

**Table 6:** Results of tests on material properties using Digimat FE for PP/PET/Cotton.

DIGIMAT FE		
PP/PET/Cotton	60/0/40	50/25/25
Modulus of elasticity E_11_/E_22_/E_33_ [MPa]	470/477/482	509/5121/556
Poisson’s ratio µ_12_, µ_23_, µ_31_	0.27/0.26/0.26	0.28/0.28/0.26
Modulus of elasticity in shear G_12_, G_23_, G_31_ [MPa]	180/183/181	207/210/211
Density ρ [kg·m^−3^]	442	521

**Table 7:** Results of tests on material properties using Digimat FE for PP/PET/glass fibers.

DIGIMAT FE			
PP/PET/glass fibers	60/0/40	50/25/25	50/45/5
Modulus of elasticity E_11_/E_22_/E_33_ [MPa]	1809/1360/2302	1445/1360/1472	620/529/553
Poisson’s ratio µ_12_, µ_23_, µ_31_	0.27/0.25/0.23	0.27/0.25/0.24	0.32/0.29/0.25
Modulus of elasticity in shear G_12_, G_23_, G_31_ [MPa]	854/962/771	579/577/576	247/231/241
Density ρ [kg·m^−3^]	640	651	527

## Data Availability

The data presented in this study are available upon request from the corresponding author due to privacy.
